# Similar social presence effects when reaching for real and digital objects

**DOI:** 10.1371/journal.pone.0232409

**Published:** 2020-05-01

**Authors:** Jill A. Dosso, Katherine Nga Tsit Chow, Jane J. Kim, Natalie T. W. Wong, Alan Kingstone

**Affiliations:** Department of Psychology, University of British Columbia, Vancouver, BC, Canada; Daegu University, REPUBLIC OF KOREA

## Abstract

Though digital images and real objects are represented differently at a neural level and can evoke different behaviours, little work has directly compared the magnitude of social effects on real and digitally represented stimuli. Object-directed reaches are modified in the near space of others, while image-directed reaches are not, but the exact role of the presence versus location of the other person is unknown (Dosso and Kingstone, 2018). The present work probed the unique contribution of social presence (a passive observer) in shaping object- and image-directed reaching behaviour. In a shape-matching game, movements were performed more slowly and less efficiently when participants were observed by the experimenter, regardless of whether participants handled real objects or digital images. Our finding that social presence affects real- and image-directed reaches similarly supports the continued use of computer-generated objects to approximate human behaviour towards real objects when social effects on object-directed actions are studied.

## Introduction

Similar social presence effects when reaching for real and digital objects Imagine sitting down to make an audio-only call from your computer. How would you feel if you realized partway through the conversation that, unbeknownst to you, your webcam was turned on and the other person could see you, and all the things that you had been doing including combing your hair, putting on makeup, eye-rolling in response to a silly comment, and so on? This embarrassing scenario reveals a fundamental truth about human behaviour: the actions we do when we think no one can see us are often modified to be more socially acceptable when we are observed.

The presence of a passive observer, while a relatively minimal source of social presence, has long been known to interact with performance in a task-dependent manner: facilitating the fast and accurate completion of simple tasks while impairing performance of complex activities [1]. The set of behaviours that are sensitive to such social presence effects is diverse, ranging from relatively simple actions like quickly turning a crank [2] or making button-press responses to minimal stimuli [3–5] up to more complex actions like gaze behaviour directed at pictures of people in social or erotic contexts [6–8] or managing food intake and purchasing decisions [9–11]. In addition to these changes in behaviour, the presence of nearby, watching strangers can be sufficient to alter the mental representation of peripersonal space, which is the space immediately around the body in which objects are reachable [12,13–15].

While a growing body of work has considered the impact of real or represented (i.e., implied) social presence, very little work has directly compared the magnitude of social effects on actions directed towards real and digitally represented stimuli. This is the case despite the fact that images and real objects are represented differently at a neural level [16–19] and can evoke different behaviour [20–25]. What we do know is that the presence of real people can affect behaviour directed towards computerized stimuli [3,5,14,26–34]. However, such work often implicitly assumes that the results obtained using two-dimensional stimuli will generalize to real object-directed actions as well. But it is unknown whether these two stimulus types are in fact equally sensitive to social presence effects.

Some caution is warranted in generalizing between behaviour directed towards real objects and images. In recent work, we asked participants to play a shape-matching game which involved making a number of reaches to tabletop objects in a freely selected sequence. Participants performed this task in one of four arrangements, crossing the factors of object realness (reaching either for real objects or for digital objects presented on a touchscreen) and social proximity (a passive observer was either located directly in front of them or off to one side). We found that their willingness to reach near to others was dependent on the nature of the reached-for object; participants delayed reaching for real but not digital objects when that reach placed their hand in close proximity to the other individual [12]. This suggests that the space near other individuals is treated as special, at least when real objects are involved. However, as there was always another individual present in the Dosso and Kingstone (2018) study, it is unknown whether real objects are more sensitive than digital objects to social presence alone, i.e. when another person is present but located outside the peripersonal space of the participant. Disentangling the potential contributions of social presence (in the form of a passive observer) and proximity on object- and image-directed actions is essential for methodological, theoretical, and applied reasons as well. Methodologically, it is of critical importance to determine what test conditions will allow for the collection of valid data with computerized images standing in for real world objects. Studies of social effects that rely on computerized stimuli often produce results that are small or inconsistent [33,35]. If these studies underestimate the true size of these effects for real objects, this would clearly be important to know. Theoretically, our present study opens the door to understanding how social presence (or absence) may lead to shared or divergent neural representations of real and digital objects. In terms of applications, our work is relevant for designers of new technologies who wish to predict how users might interact with novel items based on previously characterized social behaviours.

### Present investigation

In order to determine whether object- and image-directed reaches are equally sensitive to the presence of an observer, we compared reaching behaviour across four groups of participants, crossing the factors of object realness (real objects versus digital objects that were presented via touchscreen) and social presence (the physical presence or absence of the experimenter as an observer). Following Dosso and Kingstone (2018) we asked participants to perform a semi-structured task that would elicit multiple reaches towards the array of objects [12]. This task was selected because it can be presented in a similar manner for both digital images and real objects, and because it has been used in previously similar work examining social dynamics and technology [14,25,36,37]. Given that reaches to real objects were more sensitive to social proximity than were reaches to images of objects [12], we predicted that social presence effects would be greater for real objects than for images of objects. In accordance with our prior work, the sequence of reaches as well as their spatial and temporal profiles were measured and compared as a function of whether the objects were real or images, and whether the participants performed the task alone or in the presence of the experimenter. It is important to compare both the spatial and temporal characteristics of reaching because these speak to different potential mechanisms by which social presence might act. For example, social factors are known to change in peripersonal space representation [12,14] as well as the timing of task performance [3].

## Materials and methods

Ethics approval for this study was obtained from the University of British Columbia's Behavioural Research Ethics Board (BREB). Participants were recruited from two sources: (1) a pool of undergraduate students enrolled in Psychology courses and participating for course credit, and (2) a pool of members of the public, recruited via posted flyers, who were paid $5 for their participation.

### Task overview

In a between-subjects design, participants were randomly assigned to one of four groups based on a 2 x 2 design involving either Observed or Unobserved social presence conditions and interaction with either a touchscreen (Image group) or wooden tiles (Real Object group). Specifically, in the final sample there were four different groups of subjects: Observed-Image (*n* = 41), Unobserved-Image (*n* = 38), Observed-Real (*n* = 40) and Unobserved-Real (*n* = 40). The task was a modified version of the children’s game Memory (or Concentration) involving 24 tiles (depicting 12 pairs of animals) that are initially placed face-down. Participants were instructed to reach for items only with their dominant hand, returning to touch a designated location in front of themselves between movements for consistency of coding. Participants were instructed to flip over and reveal items in pairs. If the items matched, they were left face-up and gameplay proceeded. If they did not match, both items were to be flipped down before any new items were revealed. The game continued until all items were face-up and therefore all pairs had been found. Participants were instructed to play in a way that minimized the total number of moves used. In the Observed conditions participants were told that the (female) experimenter, who was approximately one metre away and away from the participants’ direct line of gaze, would keep count of their number of moves. In the Unobserved conditions, participants were provided with the instructions and then left alone to complete the task. In all cases, participant behaviour was unobtrusively video-recorded, and participants were notified of this recording during the debriefing process. After gameplay, participants completed a short questionnaire about the session as well as a handedness questionnaire [38].

### Image group

#### Participants

In this group, 84 participants were tested. Participants were excluded due to failure to touch the correct starting location on more than 10% of reaches (*n* = 3) or failure to select items in pairs as instructed (*n* = 2). Among the 79 included participants, self-reported sex was female (*n* = 53), male (*n* = 25), and unreported (*n* = 1). Participant handedness was classified as right-handed (*n* = 62), ambidextrous (*n* = 10), and left-handed (*n* = 6) [38]. Handedness data was missing due to an error in data collection for one participant. Included participants’ self-reported ethnicities were Asian (*n* = 57), Caucasian/White (*n* = 14), Latin American/Hispanic (*n* = 3), Middle Eastern (*n* = 3), Mixed Ethnicity (*n* = 1), and unreported (*n* = 1). Mean age was 21.7 years (*SD* = 4.5). Within these included participants, 41 had been randomly assigned to the Observed group and 38 to the Unobserved group. These two subgroups were comparable in age, sex, handedness, and ethnic composition.

#### Image task

**Fig 1:**
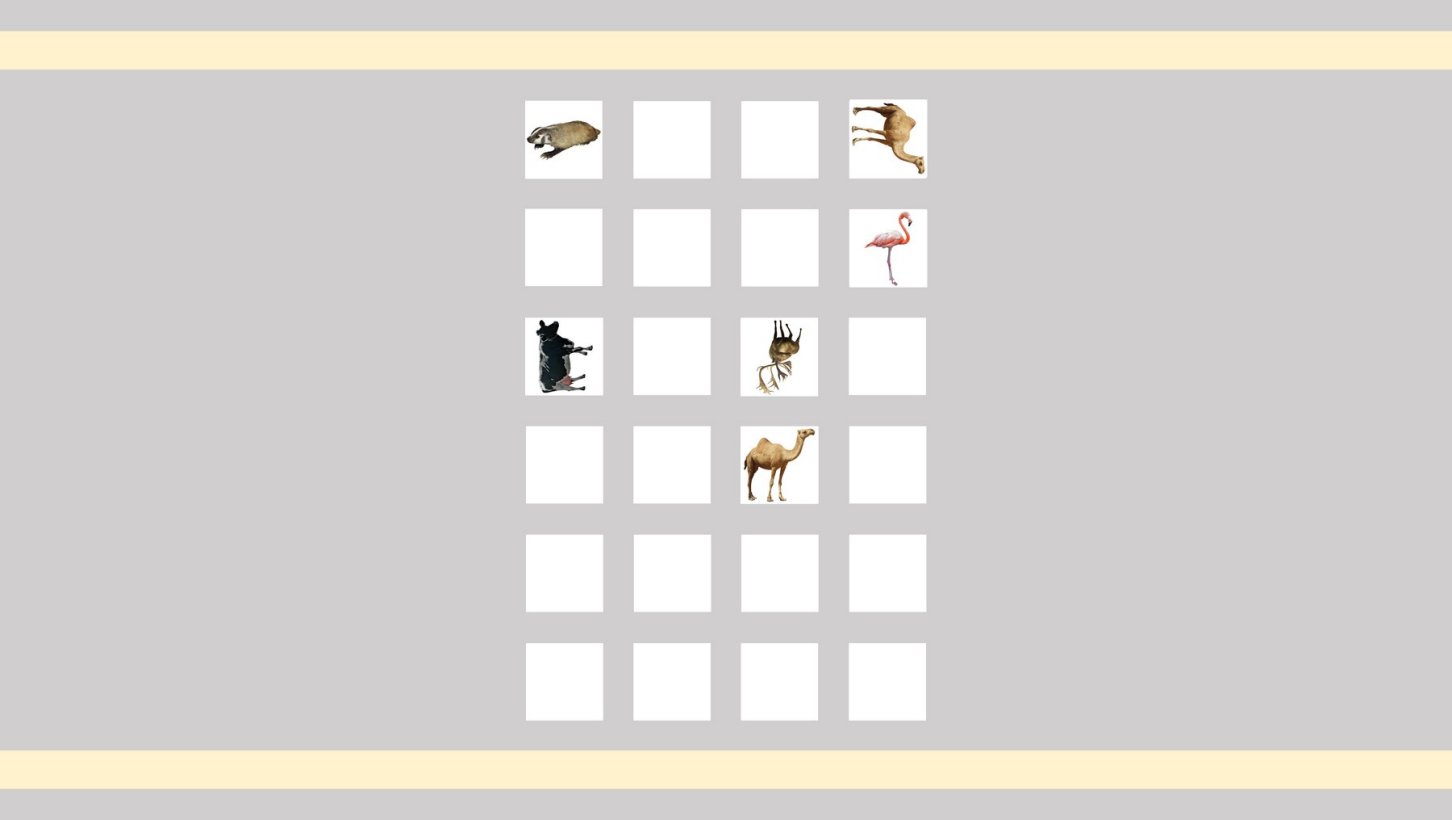
Sample screen during gameplay. The experimenter was present at a short distance for Observed participants and absent for Unobserved participants.

The task was programmed using PsychoPy [39]. The virtual tiles contained images of animals taken from the Bank of Standardized Stimuli [40]. The touchscreen itself measured 64 cm wide x 105 cm long. The 24 items (four columns and six rows) were each 5 cm square and together covered an area 26 cm wide and 44 cm long (Fig 1). In the Observed condition the experimenter remained in the room during testing and watched the participant perform the task. In the Unobserved condition the experimenter delivered the instructions and then left the room during task performance. Timing data for each object and line touch were automatically recorded by the task software.

### Real object group

#### Participants

In this group, 86 participants were tested. Participants were excluded due to technical issues in testing (*n* = 2), failure to touch the correct starting location on more than 10% of reaches (*n* = 2), and being aware of the recording procedure (*n* = 1). For the included 81 participants, self-reported sex was female (*n* = 67) and male (*n* = 14). Participants’ handedness was classified as right-handed (*n* = 72), left-handed (*n* = 6), and ambidextrous (*n* = 3) [38]. Participants reported their ethnicities to be Asian (*n* = 64), Caucasian/White (*n* = 12), First Nations (*n* = 2), Middle Eastern (*n* = 1), Hispanic/Latin American (*n* = 1), and unreported (*n* = 1). Mean age was 21.2 years (*SD* = 6.1). Within the included participants, 40 experienced the Observed condition and 40 experienced the Unobserved condition. The two subgroups were similar in their age, sex, handedness, and ethnic breakdown.

#### Real-object task

The experimental table was 89 cm long and 74.5 cm long wide. Tiles were initially placed with a blank face up, and they featured brightly coloured cartoon animals on the reverse face. Each item measured 5 cm square, and items were evenly spread to cover an area 58.5 cm long (across the table) and 37 cm wide (left-to-right, from the participant's point of view). Between each participant and their array was a red line which they were instructed to touch between each reach. In the Observed condition the experimenter remained in the room during testing and watched the participant perform the task. In the Unobserved condition the experimenter delivered the instructions and then left the room during task performance.

#### Real-object data processing

All real-object sessions were inconspicuously video-recorded using a laptop placed on a desk to the side of the experimental table to enable later coding of reaching sequence. The laptop screen was dark and all computer lights were off. No included participants reported knowledge of being recorded. Object touches and time taken from each line touch to each subsequent object touch (“turn duration”) was hand-coded from video recordings by two trained coders. Note that turn duration does not include the additional time taken to rotate and handle the physical objects; it was defined in this way to make comparison between real object and image conditions as straightforward as possible. Coder 1 coded 7 videos alone, coder 2 coded 63 videos alone, and 11 videos were coded independently by both coders. Coding reliability for turn duration was high, Cronbach’s ɑ = 0.98. Agreement for the row of each reach was 97.4%. Coders were blind to the goals of the study. Data analysis was performed in JASP 0.10.2 [41].

## Results

All data are freely available at https://osf.io/zvpts/.

### Total moves

Total moves were counted for each participant (including the moves on which they touched the wrong start bar, which were excluded for RT-based analyses). On average, participants made 56.4 reaches (*SD* = 13.6). Total moves were subjected to an ANOVA with condition (observed or unobserved) and task (real object or image) as between-subjects factors. This analysis produced no main effects or interactions (all *p*>.50, ƞ_p_^2^ < .01); total moves taken to perform the task was not different across groups.

### Reaching sequence

**Fig 2:**
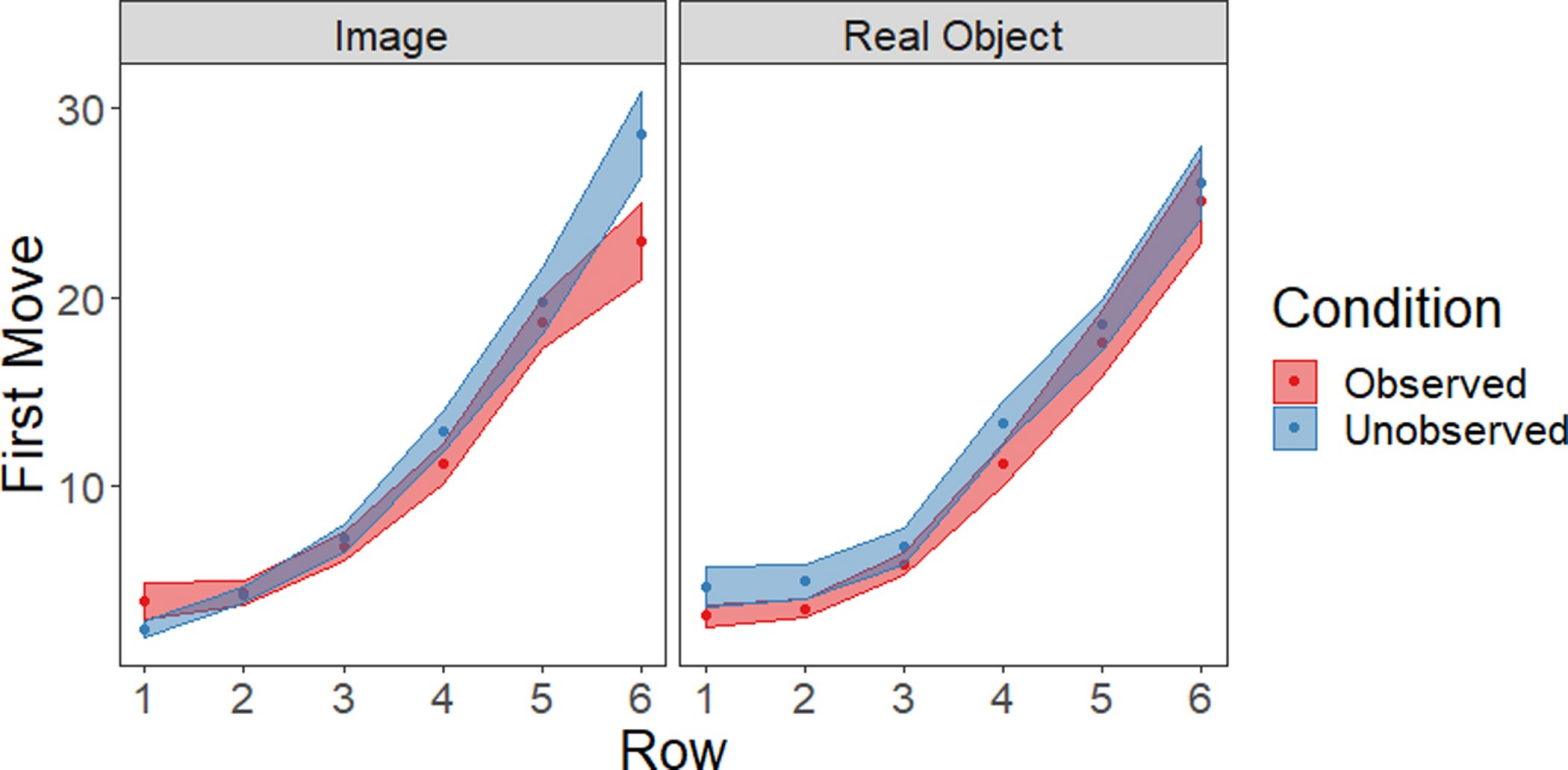
Reaching sequence to enter new rows of the board. Mean ± SE of first moves are shown.

For each participant, each reach to reveal an object (even if that object had been revealed unsuccessfully before) was numbered. The move number on which each row was contacted for the first time was identified (Fig 2). For example, if a participant’s first reach was to an object in Row 2 and their second reach was to an object in Row 1, the first move for Row 2 was move number one, the first move for Row 1 was move number two, etc. These move numbers were then subjected to a repeated-measures ANOVA with Row as a within-subjects factor, and social condition (observed or unobserved) and stimulus type (real object or image) as between-subjects factors. This analysis produced a main effect of Row (*F*(5, 780) = 236.1, *p* < .001, ƞ_p_^2^ = .60); more distant rows were contacted later in the action sequence than more proximal rows. There was a non-significant effect of social condition (*F*(1, 156) = 3.1, *p* = .08, ƞ_p_^2^ = .02); new rows were contacted marginally later in gameplay when participants were unobserved. There were no additional main effects or interactions (all *p*>.25, ƞ_p_^2^ < .01).

### Turn duration

**Fig 3:**
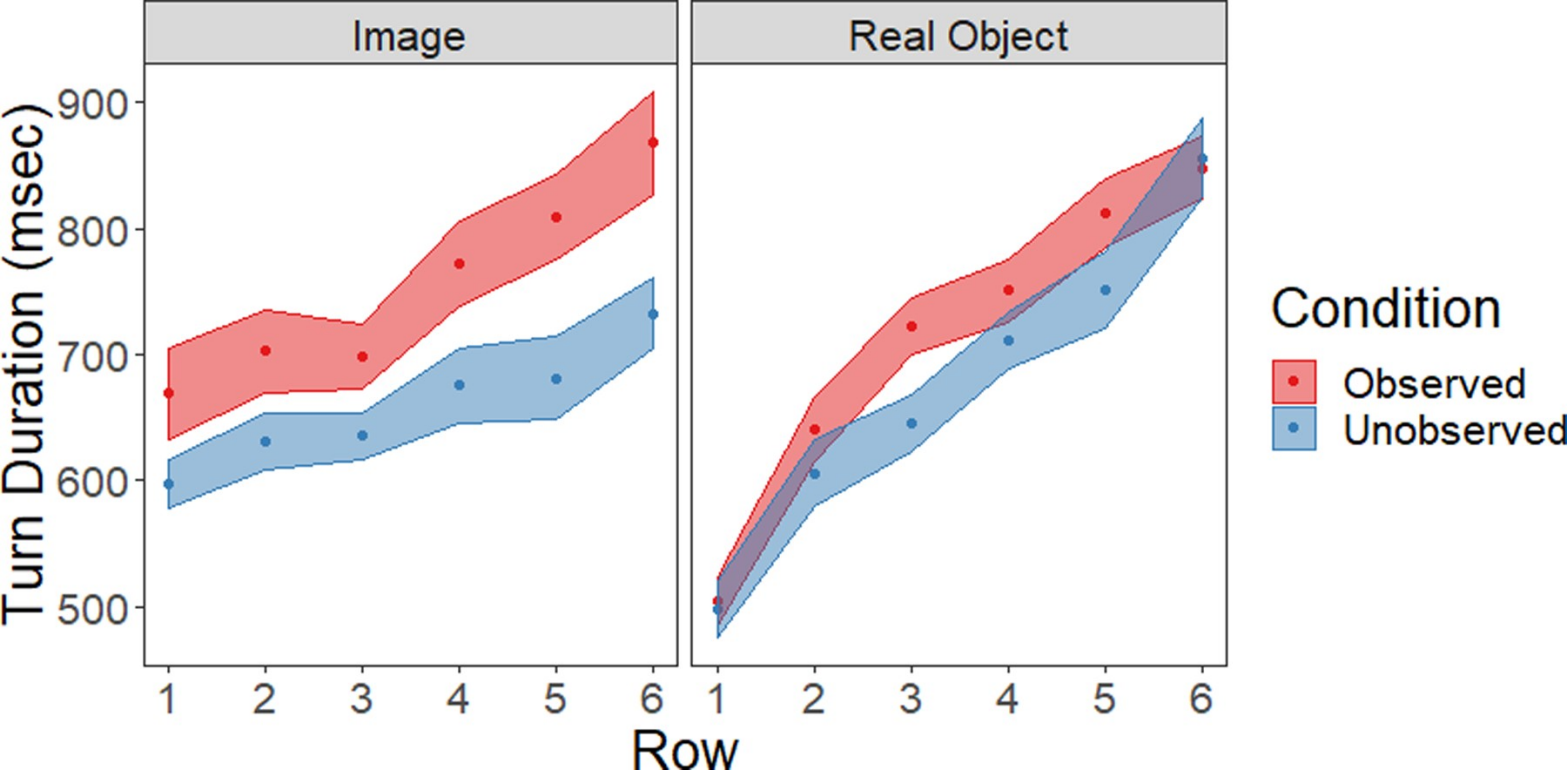
Turn duration across the array in milliseconds (mean ± SE).

A turn was coded as the time taken from touching the line in front of the participant to touching the next object (Fig 3). Turn durations were excluded for each participants’ first action of the testing session, if the participant failed to touch the line in front of themselves prior to reaching, and if their length exceeded 2 SD from the mean turn duration for that participant reaching into that particular row. Cleaned turn durations were subjected to a repeated-measures ANOVA with row as a within-subjects factor, and social condition (observed or unobserved) and stimulus type (real object or image) as between-subjects factors. This analysis produced a main effect of row (*F*(5, 780) = 77.9, *p* < .001, ƞ_p_^2^.33); unsurprisingly, turns took longer when more distant objects were contacted. There was also a row by stimulus type interaction (*F*(5, 780) = 10.7, *p* < .001, ƞ_p_^2^ = .06); the relationship between row and increased turn duration was steeper for real objects than for images. Finally, there was a main effect of social condition (*F*(1, 156) = 10.0, *p* = .002, ƞ_p_^2^ = .06); observed participants took longer turns than unobserved participants regardless of stimulus type. There were no additional main effects or interactions (all *p*>.15, ƞ_p_^2^≤.01).

### Performance errors

**Fig 4:**
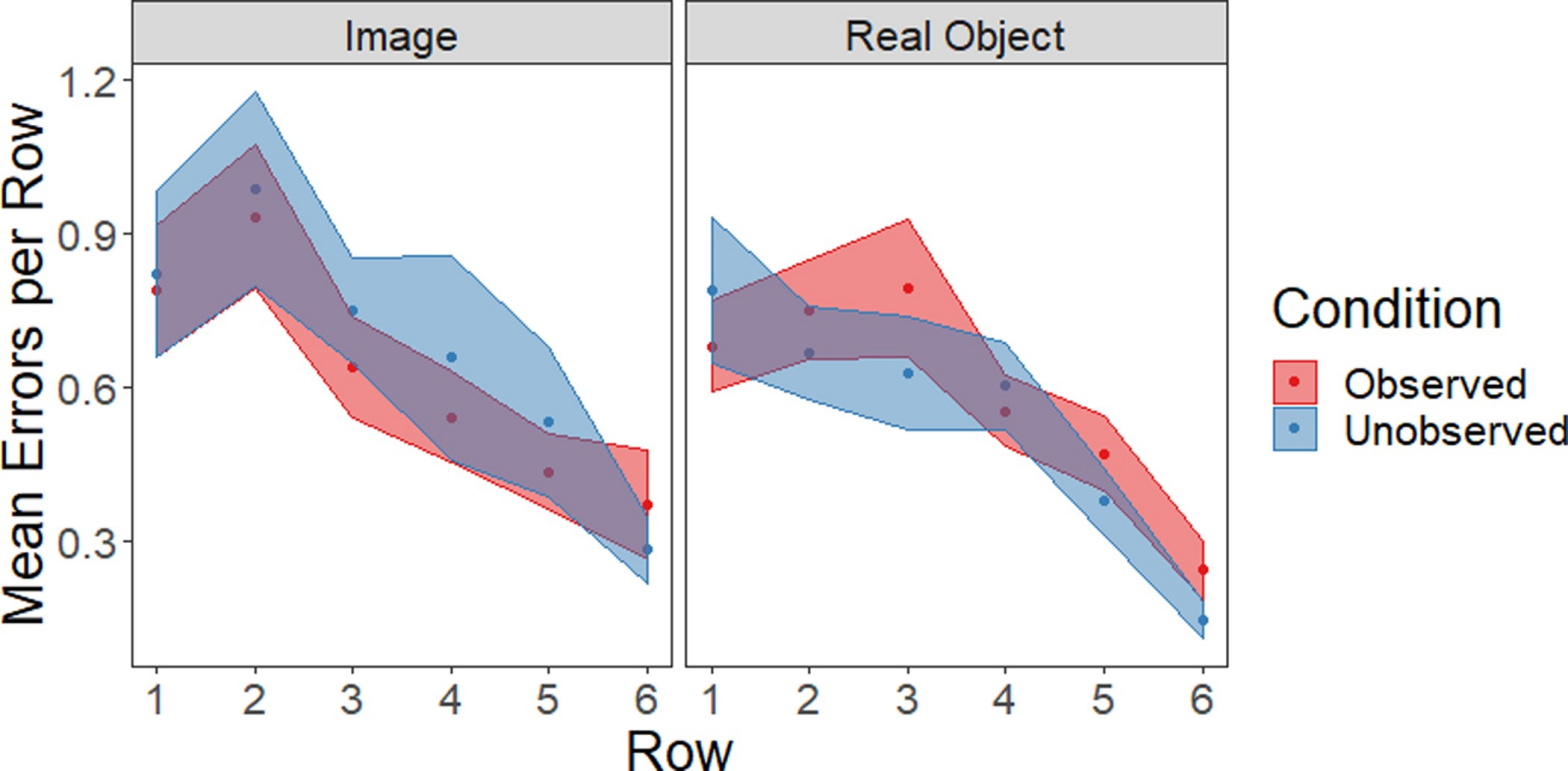
Errors made in the task (mean ± SE shown).

Next, we examined the success of participants’ gameplay (Fig 4). To do this, we identified each move for which a particular object was seen for neither the first nor the last time. In essence, these are moves on which the item has been seen before (so the opportunity to store the item in memory has occurred) but on which the item is not successfully matched. The number of errors made for each row was calculated for each participant, and a repeated-measures ANOVA was conducted with row as a within-subject factor and social condition (observed or unobserved) and stimulus type (real object or image) as between-subjects factors. We observed a main effect of row (*F*(5, 780) = 23.9, *p* < .001, ƞ_p_^2^ = .13); fewer errors were made in more distant rows. There were no additional main effects or interactions (all *p*>.25, ƞ_p_^2^ < .01).

### Efficiency scores

**Fig 5:**
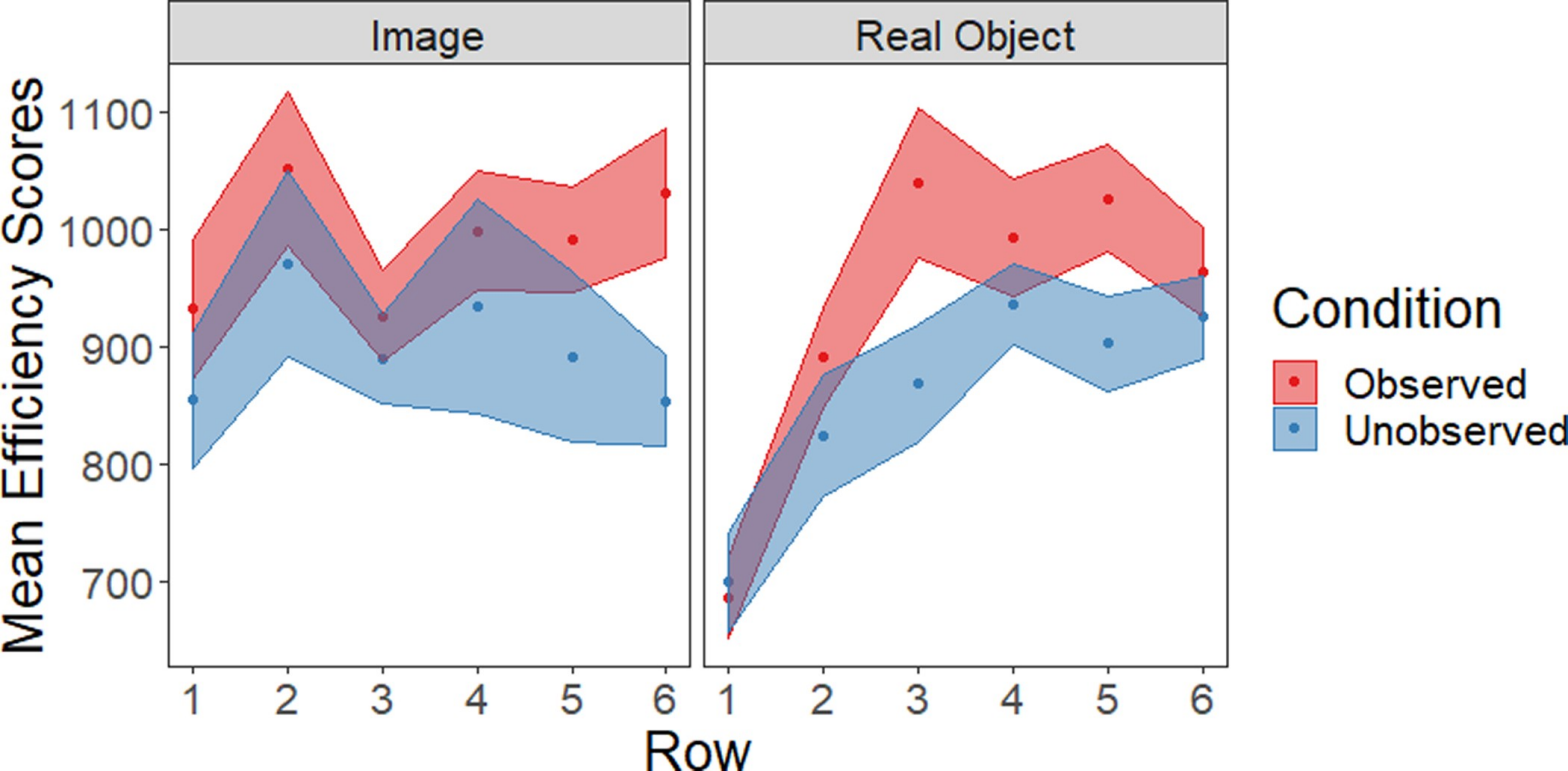
Efficiency scores for this task (mean ± SE shown).

Next, we calculated efficiency scores (Fig 5) to evaluate whether the data were characterized by speed-accuracy trade-offs. Efficiency scores were calculated as mean turn duration per participant per row, divided by the proportion of reaches that were not errors (i.e. where the item in question had either never been seen before or was successfully matched). Smaller efficiency scores indicate more efficient performance [12]. A repeated-measures ANOVA was carried out, as before. Effects matched those seen for turn duration: efficiency scores were higher (indicating slower/less efficient performance) for more distant rows (*F*(5, 780) = 10.2, *p* < .001, ƞ_p_^2^.06), efficiency scores were more tightly linked to location for real objects as indicated by a row by object type interaction (*F*(5, 780) = 6.7, *p* < .001, ƞ_p_^2^.04), and observed participants were less efficient than unobserved, shown as a main effect of social condition (*F*(1, 156) = 4.6, *p* = .03, ƞ_p_^2^ = .03). There were no additional main effects or interactions (all *p*>.20, ƞ_p_^2^≤.01).

## Discussion

Researchers often use computerized image-based tasks to study social behaviour in the lab. This is done with the implicit assumption that these tasks also capture the same social influences that would be present for real object-directed actions [3,5,14,26–34]. However, this assumption has recently been called into question by the finding that image-directed reaches made on a touchscreen are less sensitive to a social manipulation (interpersonal spacing) than real object-directed reaches [12]. It is common for digital image-directed actions to be studied by researchers and technological innovators in the presence of an observer, i.e. a source of social presence [5]. Therefore, the present work asks: are actions directed towards real objects and towards digital images equally sensitive to social presence effects?

We manipulated this via the simple presence or absence of the experimenter during testing. This type of “mere presence” has been shown to be sufficient to change the speed and accuracy of some types of task performance [1], and these effects tend to be more pronounced when the observer is the experimenter [42]. We chose this manipulation because there are known impacts of social factors on both real [12,43,44] and computerized objects [5,31,34] and we intended to make a direct comparison of such effects across the two stimulus types. Furthermore, we considered potential social effects in two categories: spatial changes in behaviour, and temporal changes.

Spatially, all participants showed a general tendency to first select objects near themselves and to work their way outward as the game progressed (Fig 2).This pattern is typical in tasks like these [14,36]. Our past work found that participants’ hesitancy to reach into the space around a peer’s body was related to the nature of the objects that were touched: they showed a greater delay in reaching for real objects near others than for digital images [12]. Facing nearby others has been shown to shrink the size of one’s peripersonal space [13,15] and therefore we had hypothesized that this might modulate object- but not image-directed actions. However, we were unable to assess what effect social presence alone has on actions to real versus digital objects because our study lacked a “no social presence” baseline condition.

The current study addresses this issue by manipulating social presence while controlling for any effect of peripersonal space by ensuring that participants never execute a reach near others. We found no differences in the reaching sequences used by participants based on the presence or absence of an observer. This was true regardless of whether the items being handled were real objects or digital images. Similarly, there were no effects of the social and stimulus manipulations on the spatial distribution of participants erroneous moves. By extension this suggests that the social proximity effect in Dosso and Kingstone (2018) was a location-specific effect; when participants avoided object-directed reaches in the space near a peer, it was due to a space-specific mechanism like peripersonal space contraction, not a general social presence effect.

In terms of timing, we found that movements were performed more slowly and less efficiently when participants were observed by the experimenter (Fig 3, Fig 5). This is consistent with typical social presence effects in which complex actions are performed more slowly in social contexts [1,43,45]. Importantly, this effect of social presence was not different when participants handled real objects and virtual images. Such a non-spatial effect is likely to occur via mechanisms including increased conformance to social norms (i.e. moving more slowly in an attempt to increase accuracy) due to increased self-awareness, self-presentation concerns, or an increased tendency to engage in social comparison [3,42,46,47].

The finding that non-spatial social presence effects manifested similarly across real and digital items provides support for researchers’ long-standing assumption that computerized objects can, in some cases, adequately stand in for real objects without the loss of naturalistic social dynamics. Our conclusion is that, at least when an observer is not in the reachable space of another, reaches for real and digital objects are comparable. It is reasonable, then, for designers of new technologies to predict that, in similar contexts, effects obtained with digital objects are likely to apply to real objects. Of course, this does not preclude the possibility that other, more powerful social dynamics may play out differently for real and digital objects. For example, subjects approach virtual others most closely than real others, and gaze at digital representations of people differently than real people [48,49] It is therefore important for future work to continue to unbraid the intertwined roles of the physical and social environments on human action and performance.
